# Nutrition and the gut microbiome: a symbiotic dialogue influencing health and disease

**DOI:** 10.3389/fnut.2026.1761992

**Published:** 2026-02-12

**Authors:** Sayantanee Ray, Prakash Shankaran

**Affiliations:** Virology and Ayurgenomics Laboratory, SASTRA-IKS Center for Ayurgenomics, School of Chemical and Biotechnology, SASTRA Deemed to Be University, Thanjavur, India

**Keywords:** food, metabolome, metagenome, microbiome, nutrition

## Abstract

The gut microbiome, a complex consortium of trillions of microorganisms, significantly influences human health through its metabolic activities, immune modulation, and interaction with the nervous system. Diet plays a significant role in shaping the gut microbiome, with plant-based diets promoting the colonization of beneficial bacteria and fiber fermentation, whereas meat-based diet may encourage harmful microbial shifts associated with systemic inflammation. Gut bacteria produce short-chain fatty acids (SCFAs) from dietary fibers and those are crucial for energy metabolism, intestinal integrity, and immune modulation. Certain neurotransmitters like GABA and serotonin produced by gut bacteria, play a vital role in the gut-brain axis. Dysbiosis in the gut microbiota have been linked to various psychiatric and neurological disorders like anxiety, depression, bipolar disorder, Schizophrenia, Alzheimer’s and Parkinson’s. Beyond neurological implications, the gut microbiota also linked to metabolic and cardiovascular diseases, including obesity, hypertension, and coronary artery disease, as well as colorectal cancer. Imbalances in bacterial ratios, such as Firmicutes to Bacteroidetes, can impact metabolism and inflammation. This review (i) elucidates the complex interplay between nutrition and the gut microbiome, emphasizing its implications for human health and disease; (ii) critically examines the methodological and analytical limitations inherent in current metagenomic studies; and (iii) proposes an integrated, multi-layered, systems-level framework for developing predictive models of host–microbe interactions and their pathological significance.

## 1 Introduction

The human gastrointestinal tract harbors an exceptionally dense population of microbial cells, collectively referred to as the gut microbiome (1). This complex ecosystem plays a crucial role in maintaining health and facilitating various physiological processes. The gastrointestinal (GI) tract serves as one of the largest interfaces-spanning approximately 250–400 m^2^, between the host, external environmental factors, and endogenous antigens. Throughout an average lifespan, the GI tract processes more than 60 tons of food and is exposed to a substantial influx of environmental microbes that can challenge the integrity of the gut barrier (2). The term “gut microbiota” encompasses the diverse community of bacteria, viruses, fungi, and algae residing in the GI tract. Over millennia, these microorganisms have co-evolved with their hosts to establish a sophisticated and mutually beneficial symbiotic relationship (3, 4). Recent studies estimate that microbial cells in the human body exist in a roughly 1:1 ratio with human cells. Remarkably, the gut microbiome contains approximately 3.3 million non-redundant microbial genes which is 150 times greater than the 21,000 genes encoded by the human genome (5). In adults, the gut microbiome comprises over 1,000 distinct species and more than 7,000 bacterial strains (6). Notably, while humans share 99.9% genetic similarity with one another, their gut microbiomes can differ by as much as 80%–90%, highlighting its dynamic and individualized nature (7, 8). Diet plays a crucial role in maintaining the composition, function and diversity of the gut microbiome; eventually, gut microbiome derived serum metabolites show beneficial effects on health; while some may serve as disease biomarkers. Understanding the interplay between diet, gut microbiome and serum metabolome and its impact on health and disease will be discussed further.

## 2 Dietary source influencing gut microbiome

### 2.1 Food

**FIGURE 1:**
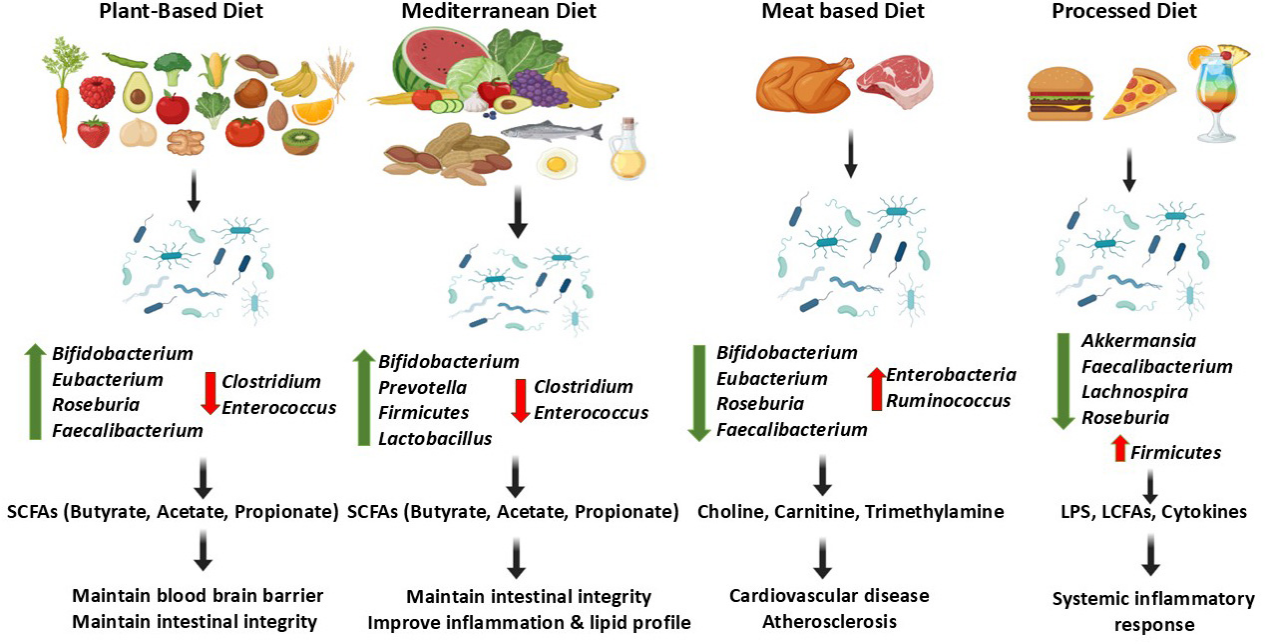
Gut microbiota derived metabolites and their function in health and disease.

Dietary habits have a profound impact on our gut ecosystem. Diet contributes macronutrients (carbohydrate, protein, fat), micronutrients (minerals and vitamins) and phytochemicals. Carbohydrate, fiber, protein and fat show significant influence on regulation of prevalence of different microbial species (9). Figure 1 showcases how gut microbiota derived metabolites affect health and disease.

#### 2.1.1 Carbohydrates

Carbohydrates can be broadly categorized into two types: digestible and indigestible. The gut microbiota ferments insoluble dietary fibers and indigestible polysaccharides into bioactive metabolites known as short-chain fatty acids (SCFAs). Among these SCFAs, acetate (C2), propionate (C3), and butyrate (C4) are the most abundant, typically found in ratios of approximately 3:1:1 (10). These linear monocarboxylic acids with fewer than six carbon atoms play critical roles in energy metabolism, maintaining intestinal barrier integrity, modulating immune cell development, and exhibiting anti-inflammatory properties (11–13). SCFA production involves multiple biochemical pathways; acetogenic bacteria utilize the Wood–Ljungdahl pathway to convert CO_2_ into acetyl-CoA directly, which can further be converted to acetate; while glycolysis converts glucose to pyruvate which can be further processed to produce acetate (14). Butyrate synthesis occurs via the transformation of aceto acetyl-CoA into butyryl-CoA and subsequently into butyrate. Acetate is predominantly synthesized by species such as *Acetobacter* and *Gluconobacter* (12, 14, 15). Key human gut bacteria that produce propionate include members of the genus *Bacteroides* (*B. fragilis*, *B. thetaiotaomicron*, *B. eggerthii*), while *Veillonella parvula* utilizes succinate as a substrate for propionate production. Additionally, *Coprococcus* and *Ruminococcus* species can produce propionate through acrylate and propanediol pathways respectively (16, 17). Within the Firmicutes phylum, bacterial genera such as Ruminococcaceae, Lachnospiraceae, Erysipelotrichaceae, and Clostridiaceae are prominent producers of butyrate. Specific species like *Clostridium butyricum* and *Butyrivibrio fibrisolvens* are also key contributors for butyrate (18, 19).

The small intestine uses enzymes to break down digestible carbohydrates, such as starch, fructose, sucrose, and lactose which are metabolized to release glucose into the circulation and trigger an insulin response. Date is a rich source of dietary fiber and polyphenols. Investigation on gut bacterial changes induced by the whole date fruit extract (digested date extract; DDE) and its polyphenol-rich extract (date polyphenol extract; DPE) revealed that both the DDE and DPE extracts enhance the gut health by increasing the beneficial bacterial growth and inhibiting the colon cancer cells. Though both extracts were found to be beneficial the DDE significantly increased both *Bifidobacteria* and *Bacteroides* levels when compared to DPE. In addition, bacterial metabolism of whole date extract (DDE) led to the production of SCFA, flavonoid aglycones (myricetin, luteolin, quercetin and apigenin) and the anthocyanidin petunidin (20). Hydrolyzed lactose has been shown to modulate the composition of gut microbiota by inducing the proliferation of *Lactobacillus* and *Bifidobacteria* while simultaneously suppressing *Bacteroides* and *Clostridia* population in infants with milk-allergy. Furthermore, metabolomic analysis elucidated that lactose supplementation significantly elevated fecal concentrations of beneficial short-chain fatty acids (SCFAs), especially acetic acid and butyric acid (21). Diets high in non-digestible carbohydrates, such as whole grains and wheat bran, have been linked to increased levels of *Bifidobacteria* and *Lactobacilli* (22, 23). The population of *Ruminococcus*, *E. rectale*, and *Roseburia* also seems to be increased by other non-digestible carbohydrates including resistant starch and whole grain barley (24–26). A cross-sectional study involving 344 patients with advanced colorectal adenomas found that, compared to healthy controls, these patients had higher levels of *Enterococcus* and *Streptococcus* but lower levels of *Roseburia* and *Eubacterium* (27). Additionally, low amount of non-digestible carbohydrates in the diet predominantly reduce the levels of *Roseburia* spp. and *E. rectale* subgroup of cluster XIVa from the Firmicutes phylum and also the level of *Bifidobacterium* from the Actinobacteria phylum (28).

#### 2.1.2 Proteins

A substantial body of research reveals a positive correlation between total microbial diversity and protein intake. Consuming whey and pea protein extract has been demonstrated to raise the population of gut-commensal *Bifidobacterium* and *Lactobacillus*, while whey protein also reduces pathogenic *Bacteroides fragilis* and *Clostridium perfringens* (29–31). Additionally, pea protein has been noted to elevate intestinal SCFA levels, which are thought to have anti-inflammatory properties and are crucial for maintaining the mucosal barrier (32). Conversely, it has been observed that the consumption of animal-based protein increases the counts of bile-tolerant anaerobes like *Bacteroides*, *Alistipes* and *Bilophila* (33–35). Increased *Bacteroides* population is associated with higher intakes of animal protein and different types of amino acids (36). A study on Italian youth found that their gut microbiota was more abundant in *Bacteroides* and *Alistipes* when they consumed higher amounts of animal protein (37). Furthermore, individuals following a high-protein, low-carb diet have lower levels of butyrate in their stools and fewer populations of *Eubacterium rectale* and *Roseburia* in their gut microbiota (38). Ingesting red meat has been linked to the promotion of numerous microbial genera that have also been linked to elevated levels of trimethylamine-N-oxide (TMAO), a proatherogenic substance that raises the risk of cardiovascular disease (39). Tryptophan is an essential amino acid, often found in most protein rich foods, including both animal and plant protein sources like chicken, eggs, fish, milk, cheese, peanuts, seeds, soy beans (40). Tryptophan is a precursor of serotonin (5-HT) and majority of body’s serotonin is produced by gut bacteria. *Lactobacillus* and *Streptococcus* can produce serotonin directly using tryptophan synthetase enzyme. *Ruminococcus gnavus* and *Clostridium sporogenes* can convert tryptophan into tryptamine via the enzyme tryptophan decarboxylase. Gut colonizer *Clostridia* sp. can signal the enterochromaffin cells of gut to increase serotonin production from this tryptamine (41). On the other hand, *Lactobacillus*, *Bifidobacterium* and *Bacteroides* produce GABA from L-glutamate through the glutamate decarboxylase pathway (42).

#### 2.1.3 Fat

Diets rich in saturated and trans fats are likely to elevate blood total and LDL cholesterol levels, thereby increasing the risk of cardiovascular disease (43, 44). Conversely, beneficial lipids such as mono and polyunsaturated fats play a major role in reducing the chance of developing chronic illnesses. Typical western diets are predisposed to numerous health issues since they are low in mono and polyunsaturated fats and high in trans and saturated fats (45–47). In a comparative study, the scientists observed that a low-fat diet increased the amount of *Bifidobacterium* in the feces while simultaneously lowering total cholesterol and fasting glucose levels. Conversely, a diet high in saturated fat raised the presence of *Faecalibacterium prausnitzii* (48). A high-fat diet has been demonstrated to lower the concentration of *Bacteroides* species from the Bacteroidetes phylum, *Eubacterium rectale*, and *Blautia cocoides* from the Firmicutes phylum (49).

#### 2.1.4 Probiotics

Fermented foods like yogurt and cultured milk products, are a source of edible microorganisms that may help to maintain intestinal health and even cure or prevent inflammatory bowel disease (IBD) (50). They are believed to achieve this through their immune modulatory properties such as activation of anti-inflammatory cytokines such as IL-10 and IL-12 (51), and reducing pro-inflammatory molecules such as IL-8 and nitric oxide (NO) levels (52) as well as their impact on the current gut flora. Foods enriched with beneficial bacteria are known as probiotics because of their health-promoting characteristics (53–56). A randomized, placebo-controlled trial involving sixty overweight but otherwise healthy adults demonstrated significant increases in the concentrations of total aerobes, anaerobes, *Lactobacillus*, *Bifidobacteria*, and *Streptococcus* among participants who received probiotics containing three strains of *Bifidobacteria*, four strains of *Lactobacilli*, and one strain of *Streptococcus*, compared to those receiving a placebo. Additionally, these participants exhibited lower levels of triglycerides, total cholesterol, LDL cholesterol, VLDL cholesterol, and high-sensitivity C-reactive protein (hsCRP), along with reduced counts of total coliforms and *Escherichia coli*. Furthermore, probiotic treatment was associated with improved insulin sensitivity and increased HDL cholesterol levels. Notably, individuals with baseline low HDL, elevated hsCRP, and greater insulin resistance initially had fewer total *Lactobacilli* and *Bifidobacteria* but more *Escherichia coli* and *Bacteroides* (57).

#### 2.1.5 Polyphenols

The antioxidant properties of dietary polyphenols, including phenolic acids, anthocyanins, flavanols, flavones, and proanthocyanidins, are being intensively researched. Foods rich in polyphenols include wine, tea, cocoa products, fruits, seeds, and vegetables (58). Ortuno et al. investigated the effect of moderate intake of red wine polyphenols on selected gut microbial groups. Daily intake of red wine for 4 weeks significantly boosted *Enterococcus*, *Prevotella*, *Bacteroides*, *Bifidobacterium*, *Eggerthella lenta*, *Blautia coccoides* and *Eubacterium rectale* populations alongside reduction in systolic/diastolic blood pressure, triglycerides, total HDL (high density cholesterol) and C-reactive protein. Notably, shifts in cholesterol and CRP correlated with *Bifidobacteria* abundance (59). However, these findings from red wine polyphenols must be interpreted cautiously, as alcohol consumption-even moderate carries substantial risks including cancer, as per the 2025 U.S. Surgeon General’s Advisory, where benefits remain uncertain and may not outweigh harms. In a study examining their antibacterial effects, enteropathogens such as *Staphylococcus aureus* and *Salmonella typhimurium* were found to be highly sensitive to polyphenols derived from fruits (60). A decrease in pathogenic *Clostridium* species has been observed with the ingestion of polyphenols from fruits (61), cocoa (62). Study on tea phenolics repressed certain pathogenic bacteria like *Clostridium perfringens*, *Clostridium difficile* and *Bacteroides* spp. while commensal anaerobe *Bifidobacterium* spp. and probiotic *Lactobacillus* sp. were less affected (63).

### 2.2 Dietary habits

#### 2.2.1 Plant-based diets

High fiber intake supports the growth of beneficial microbes and the production of SCFAs having various health benefits. Conversely, diets high in animal products are often associated with increased levels of certain pathogenic bacteria (64). Vegan and vegetarian diet rich in fruits and vegetables are commonly associated with a gut microbiome signature enriched in bacteria like Lachnospiraceae, *Butyricicoccus* and *Roseburia hominis* which specialize in breaking down SCFAs (65–68). A study investigated the Indian Thali, a traditional platter (including dal, curd, spices, sambhar and rasam) provides various kinds of phytochemicals (curcumin, flavonoids, tannins, isoflavone, anthocyanin, glucosinolates, carotenoids), is beneficial for gut microbiota (69). Anthocyanin-a subgroup of flavonoids (sub class of polyphenols) is known for its antioxidant and anti-inflammatory properties, which help mitigate oxidative stress. It reduces the accumulation of reactive oxygen species (ROS) and downregulates the expression of Interleukin 6 (IL-6) gene which upregulates a transcription factor known as STAT3. Since STAT3 drives cell proliferation and anti-apoptotic signaling, its modulation is critical; uncontrolled STAT3 activity may contribute to the pathogenesis of detrimental health outcomes such as cancer and Inflammatory bowel disease (IBD) (70). Catechin and epicatechin have anti-inflammatory and antioxidant properties. Catechins mostly found in green tea may also be useful in preventing angiogenesis and metastasis (71).

#### 2.2.2 Mediterranean diet

The Mediterranean diet is characterized by a favorable fatty acid profile, high in polyunsaturated and monounsaturated fatty acids, high intake of fiber and other low-glycemic carbohydrates, and a relatively higher intake of vegetable protein compared to animal protein (72). Significant correlations were found between the degree of Mediterranean diet adherence and elevated fecal SCFA, *Prevotella*, and other Firmicute levels. Conversely, there was a correlation between poor adherence to the Mediterranean diet and greater urine trimethylamine oxide, a marker of enhanced cardiovascular risk (39). Foods that make up the classic Mediterranean diet have also been demonstrated in several other trials to improve inflammation, lipid profiles, and obesity, potentially mediated by decreases in *Clostridium* and increases in *Lactobacillus*, *Bifidobacterium*, and *Prevotella* (73–75).

#### 2.2.3 Meat-based diets

Meat based diets have been shown to decrease the beneficial commensal bacteria like *Bifidobacterium* and *Eubacterium* spp. The Western diet regarded as high calorie fat diet contain heterocyclic amines (HCA), polycyclic aromatic hydrocarbons (PAH), and emulsifiers may lead to carcinogenesis. These compounds can damage the DNA of colonocytes and increase the risk of colorectal cancer (76). Higher meat intake is associated with increased levels of Firmicutes, *Bacteroides*, Proteobacteria, Actinobacteria (77). *Bilophila wadsworthia*, thrives on fats and bile, leads to a reduction in gut microbial diversity and an increase in harmful metabolites like TMAO, which is linked to inflammation and cardiovascular disease (78).

#### 2.2.4 Processed food

Processed food including burgers, pizza, carbonated beverages negatively impact gut microbiome by reducing microbial diversity and decreasing beneficial bacteria. *Akkermansia municiphila*, which is known to aid in weight management and improve insulin sensitivity, has been found to be less prevalent in individuals consuming diets rich in processed foods (79). One Spanish population study showed processed food consumers have decreased levels of *Lachnospira* and *Roseburia* which are commonly known as SCFAs producers; potentially harmful bacteria were elevated compared to those who consumed a diet with low processed food. These bacteria included *Blautia*, Carnobacteriaceae, Bacteroidetes which have been associated with metabolic disorders (80). Processed food contains synthetic additives, low fiber and emulsifiers including carboxymethylcellulose, polysorbate 80, carrageenan has been shown to change the microbiota, promoting pro-inflammatory microbial environment which may influence to develop obesity, Type 2 diabetes. These emulsifiers reduce *Faecalibacterium prausnitzii* which have anti-inflammatory qualities and negatively impact on the intestinal mucus layer, increasing permeability known as “leaky gut” and bacterial translocation into the bloodstream which may cause systemic inflammation (81, 82).

## 3 Gut microbiota associated diseases

**FIGURE 2:**
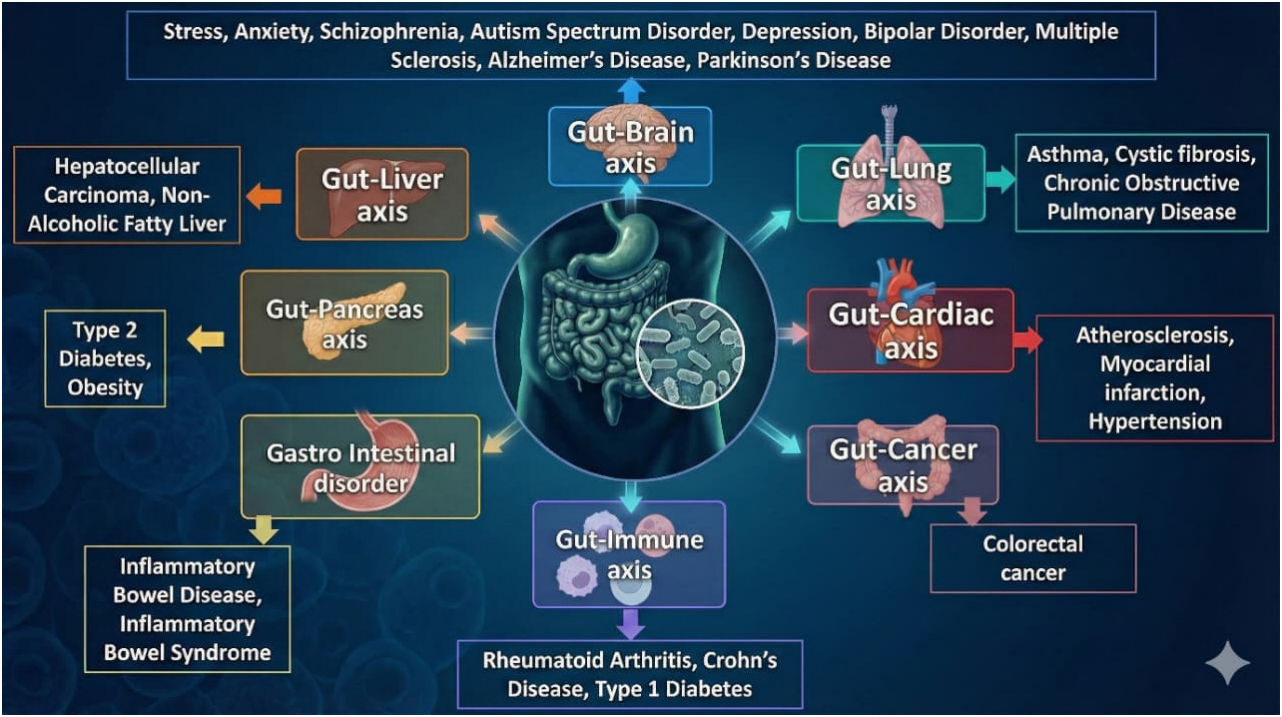
Gut-organs axis: gut-microbiome associated disease.

The gut microbiome forms complex and dynamic interactions with most of the organs of the body, establishing multiple physiological axes such as the gut-brain, gut-lung, gut-liver, gut-cardiac axis. These interconnected pathways underline the critical role of gut microbiota in maintaining systemic health and contribute to the pathogenesis of diverse diseases through disrupted microbial balance and immune modulation (Figure 2).

### 3.1 Gut-immune axis

The gut microbiota plays a critical role in the development and regulation of the host immune system through metabolites such as SCFAs contributes to host immune regulation via multiple mechanisms-including G-protein couples receptor (GPCR) signaling (e.g., GPR43/41), histone deacetylase (HDAC) inhibition and cytokine modulation-one of which involves the context dependent balance of Th17 and regulatory T (Treg) cells to support immune homeostasis in physiological states (83, 84) and diseases like rheumatoid arthritis (RA) or autoimmune hepatitis (AIH) (85, 86). The differentiation of the four primary subtypes of CD4+ T cells–Th1, Th2, Th17, and Tregs–is significantly influenced by the gut microbiome (87). Tregs, in particular, are crucial in mitigating inflammatory responses and preventing autoimmune diseases by suppressing other cell types (88). *Clostridium* species can promote Treg production (89), while *B. fragilis* can activate Tregs to inhibit pro-inflammatory Th17 responses (90). Moreover, colonic Tregs possess a unique repertoire of T cell receptors that recognize bacterial components in the colon (91). Additionally, the gut microbiota is essential for the existence and functionality of intestinal CD8+ T cells, as well as their capacity to modulate other immune cell populations, including natural killer cells, plasmacytoid dendritic cells, and marginal zone B cells (92–94).

Rheumatoid arthritis (RA) is a chronic autoimmune, inflammatory, and systemic disease characterized by the progressive degeneration of bone and cartilage, ultimately resulting in functional impairment. The inflammatory response in RA is mediated by CD4+ T cells, particularly Th1 and Th17 lymphocytes, as well as an imbalance between Th17 cells and Tregs. This dysregulation has been closely linked to the pathogenesis of RA (95). Taxonomic analyses have revealed that RA patients exhibit reduced levels of Actinobacteria compared to healthy controls. Experimental approach shows *Lactobacillus* species–such as *L. salivarius*, *L. iners*, and *L. ruminis*–predominate in the gut microbiota of RA patients compared to healthy individuals; (96) meanwhile machine learning approaches, such as the random forest method, specific microbial taxa like *Faecalibacterium*, *Collinsella*, and *Eggerthella* have been associated with RA. Notably, increased levels of *Collinsella* correlate with elevated production of alpha-aminoadipic acid, asparagine, and IL-17A which in turn influences gut permeability and exacerbates RA severity (97). Interestingly two different species of bacteria from similar genus showing contrasting effects; for example, *Prevotella histicola*, has been shown to inhibit RA development; whereas, *Prevotella copri* is more abundant in some patients with early-stage RA and has been implicated in disease progression (98). Patients with Crohn’s disease-an autoimmune disorder of digestive tract showed a reduction in *Faecalibacterium* and *Roseburia* spp. but an increase in *Escherichia*, *Fusobacterium* and *Mycobacterium* spp. (99). It was discovered that the lactate and butyrate-producing bacteria in the control group were responsible for the production of mucin, which is essential for maintaining gut integrity. Conversely, non-butyrate-producing lactate-utilizing bacteria, which are implicated in Type 1 diabetes and β-cell autoimmunity suppressed the production of mucin (100).

### 3.2 Gut-brain axis

The acquisition of gut microbiota begins at birth, and by the age of 12–36 months, infant microbiota profiles resemble those of adults (101–103). This critical period, spanning from birth to 36 months, is marked by significant neurodevelopmental milestones and immune system maturation (104–106). During this time, the gut microbiota plays an active role in various neurodevelopmental processes, including the formation of the blood-brain barrier (BBB) (107), neurogenesis (108), microglia maturation (109), and myelination (110, 111). These mechanisms play a crucial role in shaping cognitive functions and behavioral patterns. The gut microbiota produces a range of metabolites that serve as signaling molecules capable of directly stimulating the vagus nerve or crossing the intestinal barrier into the circulatory system and potentially the blood-brain barrier, thereby influencing neurological functions (112, 113). Commensal bacteria, such as Lactobacillus, synthesize neurotransmitters like gamma-amino butyric acid (GABA), dopamine, noradrenaline, and histamine, which are essential for neurophysiological and neurodevelopmental processes (114). The gut microbiome is interconnected with the central nervous system through several pathways, including the enteric and autonomic nervous systems, the hypothalamic-pituitary-adrenal (HPA) axis, and hormone signaling networks (115, 116). One such pathway involves tryptophan metabolism, which yields serotonin as a final product, primarily produced by gut flora such as *Streptococcus* spp., *Enterococcus* spp., *Escherichia* spp., and *Klebsiella pneumoniae*. The gut-brain axis, particularly its connection to anxiety and stress disorders, has been linked to the HPA axis (40, 117).

#### 3.2.1 Psychiatric disorder

Mental illness is a hidden epidemic that has been progressively spreading throughout the world. Gut microbiota dysbiosis is linked to higher risk of mental health condition and psychiatric diseases (118, 119). In a study involving 40 patients diagnosed with generalized anxiety disorder (GAD) exhibit altered gut microbiota compositions compared to 36 healthy individuals, characterized by reduced levels of Firmicutes–which are key producers of SCFAs–and increased levels of Fusobacteria and Bacteroidetes (120). Another study demonstrates patients with depression had lower levels of *Dialister* and *Coprococcus* spp. (121). The data demonstrated that major depressive disorder patients had reduced levels of Bacteroidetes in contrast increased abundances of *Prevotella*, *Klebsiella*, *Streptococcus*, and *Clostridium* XI (122). In bipolar disorder (BD), gut microbiota diversity is notably diminished, with a predominance of Clostridiaceae and *Collinsella* (123). Bipolar illness patients had much lower levels of *Faecalibacterium*, according to another cross-sectional investigation (124), alongside reduced abundances of Ruminococcaceae. One study demonstrate higher levels of Actinobacteria and Coriobacteria have been observed while (125) interestingly no significant differences were found in the counts of *Bifidobacterium*, *Lactobacillus* bacteria between the two cohorts of 39 people with bipolar disorder and 58 healthy persons (126). In children with autism spectrum disorder (ASD), gut microbiota analyses have shown increased abundances of Actinobacteria, Proteobacteria, and Bacilli (127). From an Egyptian study it has been revealed that children with ASD had higher abundance of *Clostridium paraputrificum*, *Clostridium bolteae*, and *Clostridium perfringens* compared to the neurotypical children. Notably, children with ASD harbored *Clostridium diffiicile* and *Clostridium clostridioforme*, while neurotypical children exclusively carried *Clostridium tertium*; thus, species level identification revealed clear difference between the two cohorts (128). Apart from this, there is a substantial correlation between the ratio of Firmicutes to Bacteroidetes in the gut microbiota and hypertension (129).

#### 3.2.2 Neurodegenerative disorder

Gut microbiota dysbiosis cause high permeability of the intestine and BBB through the gut-brain axis by increasing LPS, T helper cells, pro inflammatory cytokines; which results in misfolded protein accumulation, neuronal demyelination and axon damage. These can lead to neurodegenerative disorders like multiple sclerosis (MS), amyotrophic lateral sclerosis (ALS), Alzheimer’s disease (AD), Parkinson’s disease (PD) (130).

*Akkermansia muciniphila*, *Hungatella hathewayi*, *Ruthenibacterium lactatiformans* and *Eisenbergiella tayi* have been found in higher proportion in the patients diagnosed with MS while beneficial microbes that produce SCFAs like *Faecalibacterium prausnitzii* and *Blautia* sp. have been found in lower abundance (131, 132). Altered microbial structure of ALS patients has been reported, lower abundance of Firmicutes and *Megamonas* at phylum and genus level respectively while higher abundance of *Bacteroides* at the phylum level (133). *Dorea formicigenerans*, *Oscillibacter* sp., *Faecalibacterium prausnitzii*, *Coprococcus catus* were mostly associated with preclinical AD status (134). In another study 16s rRNA amplicon sequencing revealed that AD patients had a decrease in *Faecalibacterium* and increase in *Bifidobacterium* compared to controls (135). According to reports, the relative abundance of Verrucomicrobiaceae and *Akkermansia* is higher in PD patients while Prevotellaceae is lower (136). A meta-analysis of 15 case-control studies identified that Ruminococcaceae, Bifidobacteriaceae, Christensenellaceae and Verrucomicrobiaceae were enriched in PD patients. In contrast Prevotellaceae, Lachnospiraceae and *Faecalibacterium* were significantly reduced in PD patients compared to healthy controls (137). Patients with schizophrenia exhibited higher abundances of *Veillonella* accompanied by reduced level of *Ruminococcus* and *Roseburia* (138). Another study found increased prevalence of Lachnospiraceae in schizophrenia patients along with a unique microbiome composition (139). Furthermore, various facultative anaerobes, such as *Lactobacillus fermentum* and *Enterococcus faecium*, which are uncommon in healthy individuals, were identified in schizophrenic patients (140). A separate cross-sectional study revealed that schizophrenia patients had higher levels of Proteobacteria, while exhibiting reduced abundances of *Faecalibacterium* and Lachnospiraceae (141).

### 3.3 Gut-pancreas axis

A subset of gut bacteria, collectively referred to as the obesogenic gut microbiota, including certain species of Firmicutes, Bacteroidetes, *Rhizobium*, *Lactococcus*, and *Clostridium*, are positively associated with obesity development (142). Research has demonstrated that transitioning from a high-fat, low-fiber diet to a low-fat, fiber-rich diet can induce profound changes in gut microbiota composition within a mere 24 h. An elevated intake of dietary fat reduces the population of *Lactobacillus* organisms while increasing Gram-negative bacteria (143, 144). Especially, an elevated level of *Lactobacillus reuteri* and lower levels of *Lactobacillus casei* and *Lactobacillus plantarum* were correlated to obesity (145–148). In obese persons energy storage level is high due to the enhanced hydrogen transfer between microbial taxa. This is accompanied by a concurrent increase in hydrogen-producing *Prevotella* and methanogenic archaea that utilize nitrogen (149, 150). The underlying mechanism involves methanogenic archaea converting hydrogen into methane, thereby allowing for greater energy extraction from the same daily caloric intake (151–154). Patients with Type 2 diabetes mellitus (T2DM) have been reported with gut microbiota dysbiosis. Opportunistic pathogens including *Clostridium hathewayi*, *Clostridium symbiosum*, *Clostridium ramosum*, *Bacteroides caccae*, *Eggerthella lenta* and *E. coli* increased in T2DM patients while healthy controls showed higher abundance of butyrate producing bacteria (155, 156). Another study showed *Eubacterium rectale*, *Faecali prausnitzii*, *Roseburia intestinalis*, *Roseburia inulinivorans* are particularly depleted in T2DM patients (157–159). *Akkermansia muciniphila* and *Faecali prausnitzii* protect against the pathogenesis of T2DM by maintaining the integrity of mucin layer and reducing inflammation (160, 161).

### 3.4 Gut-liver axis

A targeted metagenomics and metabolomics study involving non-alcoholic fatty liver patients from Italy showed a greater level of 2-butanone and 1-pentanone and lower levels of *Oscillospira* compared to healthy controls (162). In hepatitis B virus-related liver cirrhosis (HBV-LC) *Bacteroides*, *Prevotella*, *Escherichia*, *Parabacteroides*, *Veillonella*, and *Klebsiella* were enriched while *Bifidobacterium*, *Faecalibacterium*, *Roseburia*, *Ruminococcus*, *Blautia*, *Eubacterium* were reduced in genus level. Additionally, species level identification showed enrichment of *Prevotella copri*, *Bacteroides vulgatus*, *E. coli*, *Fusobacterium nucleatum* and *Veillonella* spp. in HBV-LC (163).

### 3.5 Gut-cardiac axis

The composition of gut microbiota also differs significantly in patients with coronary artery disease (CAD), with Bacteroidetes being less prevalent and Firmicutes more abundant. One metabolite that contributes substantially to atherosclerosis and can be used to estimate cardiovascular risk is trimethylamine-N-oxide (TMAO) (164). Severe coronary atherosclerosis is known to be associated with elevated Enterobacteriaceae (165). Similarly, CAD is associated with an increased abundance of *Streptococcus* and Enterobacteriaceae (166). Heart failure patients showed increase in pathogenic bacteria in their gut microbiome; *Campylobacter*, *Salmonella*, *Shigella*, *Candida* species (167).

### 3.6 Gut-cancer axis

Imbalance in gut microbiome can lead to chronic inflammation and this sustained inflammatory response can damage DNA in cells, which may initiate tumor development. In patients with prostate cancer, elevated levels of *Bacteroides massiliensis* and significantly lower levels of *Eubacterium rectale* and *Faecalibacterium prausnitzii* have been detected, indicating these particular microbes may play a role in the etiology of prostate cancer (168). The development of colorectal cancer is associated with gut microbiota, and key contributors in this process have been identified as *Fusobacterium nucleatum*, *Bacteroides fragilis* and *Peptostreptococcus anaerobius* (169). Research suggests that gut bacteria, particularly *F. nucleatum* and *Clostridium colicanis*, are suggestive indicators of the carcinogenesis of stomach cancer (170). *F. nucleatum* can promote cellular proliferation and inhibit the host’s immunological response. Notably, a diet rich in dietary fiber and whole grains is associated with a decreased incidence of *F. nucleatum* positive cancer, suggesting that the gut microbiota may play a key mediating role in the interactions between food and colorectal cancer (171).

### 3.7 Gastrointestinal disorder

A healthy diverse microbiome helps maintain a strong intestinal barrier while dysbiosis in gut microbiome leads to “leaky gut,” allowing pathogens and food antigens to cross the barrier and trigger immune response which further leads to inflammation. In inflammatory bowel disease (IBD) patients, significant reductions in Firmicutes and Proteobacteria have been noted, including a decrease in the *Clostridium leptum* group, particularly *F. prausnitzii* (172). A study including 80 patients with irritable bowel syndrome and 65 healthy controls demonstrated that *Ruminococcus gnavus* and Lachnospiraceae species were significantly more abundant in irritable bowel syndrome (IBS); in contrast *Barnesiella intestinihominis* and *Coprococcus catus* were less abundant in IBS (173).

### 3.8 Gut-lung axis

The lung microbiota may safeguard against respiratory infections caused by *Streptococcus pneumoniae* and *Klebsiella pneumoniae* by boosting the pulmonary production of granulocyte-macrophage colony-stimulating factor (GM-CSF) via IL-17 and Nod2 activation (174). Studies on germ-free mice revealed that acute lung infections caused by *K. pneumoniae*, *S. pneumoniae*, or *P. aeruginosa* were associated with higher morbidity and mortality rates (174–176). Segmented filamentous bacteria are important for lung resistance against bacterial infections due to their ability to increase neutrophil numbers in the lungs during *Staphylococcus aureus* pneumonia and promote IL-22 and Th12 cytokine release (177). Increased gastrointestinal permeability in Chronic obstructive pulmonary disease (COPD) patients suggests the gut microbiota may contribute in worsening symptoms (178). However, the level of systemic gut microbiota-dependent TMAO has been linked to mortality, regardless of the cause of permeability (hypoxemia or pro-inflammatory state) (179). Another research discovered that early *Bacteroides* colonization, including *B. fragilis*, may serve as a precursor to asthma later in life (180).

The influence of the aforementioned dietary habits on gut microbiome diversity and abundance–and the subsequent fluctuation in microbiome-derived metabolite concentrations–demonstrates a strong correlation with disease pathology. This dynamic, conceptualized as the “Diet-Microbiome-Metabolome-Disease” axis, functions to either mitigate or exacerbate disease states. Tables 1, 2 synthesize findings from individual studies to delineate the specific protective or deleterious effects arising from these diet-microbiome interactions.

**TABLE 1:** The dietary source - gut microbiota – metabolites axis that protects against diseases.

Groups of gut metabolites	Microbial metabolites	Dietary sources	Gut microbes	Function	Protective against disease	References
Short chain fatty acids	Butyrate, acetate, propionate, 2-methyl propionate, iso-valerate, valerate, iso-butyrate, succinate	Apples, banana, pears, garlic, onion, sweet potatoes, oats, barley, millet, beans, lentils, chickpeas, soy, cheese, butter, yogurt, kimchi, kefir, almonds, flax seeds, chia seeds	*Bifidobacteria bifidum*, *Bifidobacteria infantis*, *Bifidobacteria breve*, *Faecalibacterium prausnitzii*, *Akkermansia muciniphila*, *Clostridium leptum*, *Prevotella* spp., *Veillonella* spp., *Eubacterium hallii*, *Eubacterium rectale*, *Coprococcus* spp., *Dialister* spp., *Megasphaera elsdenii*, *Roseburia intestinalis*, *Roseburia inilivorans* and *Bacteroides* spp.	Fiber fermentation, gut barrier integrity, circadian rhythms, inhibition of pro-inflammatory cytokines, improve insulin sensitivity, synthesis of vitamin B, maintain gut-brain axis, and modulation of the systemic immune response.	Protective against autism spectrum disorder, sclerosis, Parkinson’s disease, asthma, diabetes, obesity, pancreatitis, non-alcoholic fatty liver disease, hypertension, atherosclerosis, chronic kidney disease, ulcerative colitis, Crohn’s disease, colorectal cancer	(12, 66, 67, 127, 128, 137, 150, 171, 180, 182–213)
Tryptophan and indole derivatives	Kynurenine, tryptamine, serotonin, indole-3-lactic acid, indole-3-acetic acid, indole-3-acetamide, indole, indole-3-propionic acid.	Fish, salmon, poultry eggs, red meat, chicken, beef, pork, cauliflower, cabbage, broccoli, dates, chocolate, yogurt, cheese, soybeans, oats, almonds, peanuts, chickpeas, sunflower seeds, chia seeds, sesame seed.,	*Bacillus*, *Pseudomonas aeruginosa*, *Streptomyces*, *Candida*, *Escherichia coli*, *Enterococcus faecalis*, *Streptococcus*, *Clostridium* spp., *Bacteroides* spp., *Lactobacillus*, *Bifidobacterium*, *Staphylococcus*, *Peptostreptococcus*, *Lactococcus*, *Klebsiella*, *Ralstonia*, *Ruminococcus*, *Blautia*, *Citrobacter*, *Edwardsiella*, and *Providencia*	Affect the virulence, drug resistance, biofilm development, and spore formation of gut microorganisms; control the functions of the intestinal barrier, gut hormone secretion, gut motility, and systemic immune response.	Protective against irritable bowel syndrome, migraine, schizophrenia, Alzheimer’s disease, Parkinson’s disease, autism spectrum disorder, obesity	(115, 117, 214–239)
Neurotransmitter	Gamma amino butyric acid, dopamine, glutamate, catecholamine, 5-hydroxy tryptamine	Kimchi, miso, fermented food, tomato, spinach, broccoli, lentils, whole grains, sunflower seeds, soybeans, walnut, avocado, almonds, dark chocolate, fish specially shrimp, halibut	*Parabacteroides*, *Eubacterium*, *Lactobacillus brevis*, *Bacteroides*, *Bifidobacterium*, *Prevotella*, *Ruminococcus*, *Clostridium*, *Lactobacillus casei*, *Lactobacillus plantarum*, *Lactobacillus rhamnosus*, *Blautia*, *Bacteroides fragilis*	Control nervous system immunity, memory, stress reactions, and gastrointestinal motility.	Protective against Parkinson’s disease, Alzheimer’s disease	(240–258)

**TABLE 2:** The Dietary source - gut microbiota – metabolites axis that promotes diseases.

Groups of gut metabolites	Microbial metabolites	Dietary sources	Gut microbes	Function	Protective against disease	References
Choline metabolites	Dimethylglycine, acetyl choline, dimethylamine, methylamine, and trimethylamine	Red meat, salt water fish, chicken, egg, beef, pork, lamb	*Acinetobacter baumannii*, *Acinetobacter calcoaceticus* *Pelobacter carbinolicus*, *Pelobacter acetylenicus*, *Fusobacterium*, *Desulfovibrio*, *Prevotella*, *Mitsuokella*	Inhibits the production of bile acids; increases thrombosis, inflammation; influences cardiac fibrosis, hypertrophy; and aggravates mitochondrial dysfunction	Promotes obesity, atherosclerosis, diabetes, heart failure, hypertension, and non-alcoholic fatty liver disease	(213, 259–279)
Lipids	Cholesterol, triglyceride, lipopolysaccharide, phosphatidylcholines.	Processed meat like sausage, poultry, milk, cheese, yogurt, cream, butter, ghee, margarine	*Escherichia coli*, *Salmonella*, *Shigella*, *Yersinia pestis*, *Brucella abortus*, *Lactobacillus*, *Eubacterium coprostanoligenes*	Systemic inflammation is triggered by lipoprotein profiles, immunological system, and hyperinsulinemia are regulated by conjugated fatty acids; cholesterol serves as a building block for the formation of bile acids.	Promote chronic hepatitis C, diabetes, obesity, hypercholesterolemia, hyperinsulinemia, and non-alcoholic fatty liver disease.	(280–290)

## 4 Discussion and conclusion

Over the past decade, a substantial body of evidence has established the critical influence of gut-microbiota-derived metabolites, the composition of the gut microbiome, and dietary inputs on human health and disease states. Progress in elucidating host–microbiome and microbially mediated metabolic interactions, together with the advent of multi-omics platforms, is poised to significantly advance mechanistic insights into these multifaceted systems. However, existing research continues to be hampered by methodological and analytical shortcomings, frequently resulting in inconsistent or equivocal findings. Addressing these challenges will require the adoption of integrative, methodologically rigorous strategies aimed at refining current analytical paradigms, thereby enabling more robust mechanistic interpretation of gut microbiome data within the context of disease pathogenesis.

This review highlights that deciphering the complex diet-gut-microbiome-serum metabolome interplay demands advanced deep sequencing strategies beyond conventional methods. The 16S rDNA sequencing approach, though widely used, is inherently constrained by its inability to resolve microbial identity beyond the genus level and its lack of explanatory power regarding strain-specific diversity. In contrast, whole genome metagenomic sequencing provides comprehensive species and strain level insights, enabling precise characterization of microbial functions and the discovery of health-associated biomarkers. Consequently, high-throughput shotgun metagenomic sequencing becomes essential for capturing the subtle microbial variations and functional pathways that underpin human health and disease (181).

To decode the complex interplay among diet, microbiota, and serum metabolites, integrative network analyses incorporating multi-omics data genomics, proteomics, transcriptomics, and metabolomics are imperative. These multi-layered approaches facilitate a systems-level understanding, allowing the development of predictive models of host-microbe interactions and their implications in pathology. Currently, most microbiome studies predominantly explore bacterial components; however, expanding research to encompass viruses, fungi, archaea, and protists will provide a more holistic view of microbiota ecology and its influence on human health.

Furthermore, host genetic factors, including polymorphisms and epigenetic modifications, are increasingly recognized as modulators of microbiome composition and activity, underscoring the importance of an integrated host-microbe perspective. Cross-organ microbiota comparisons, such as the skin and oral microbiomes, may uncover systemic pathways through which microbial communities influence overall physiology and disease susceptibility.

Ultimately, this conceptual framework posits that dietary patterns modulate the gut microbiota, which in turn functions as a dynamic bioreactor via microbial metabolism to produce signaling molecules that manifest as serum metabolites. This diet-microbiome-metabolome axis epitomizes a central hub in human physiology, with profound implications for precision nutrition. Personalized dietary interventions, leveraging insights from multi-omics datasets, hold promise for promoting eubiosis, optimizing metabolomic profiles, and mitigating disease risk. Future research should prioritize elucidating dose-response relationships between specific dietary components, such as fiber and polyphenols, and the systemic bioavailability of beneficial microbial metabolites. Therapeutic strategies ranging from prebiotics and postbiotics to targeted microbiota modulation aim to achieve predictable, durable health outcomes throughout the human lifespan.
